# Having a history of Anorexia Nervosa: Implications for Bulimia Nervosa treatment

**DOI:** 10.1186/2050-2974-3-S1-O24

**Published:** 2015-11-23

**Authors:** Emma Dove, Stephanie Hill, Bronwyn Raykos, Anthea Fursland, Susan Byrne

**Affiliations:** 1 Centre for Clinical Interventions, Australia; 2 University of Western Australia, Australia

## 

The aim of this study was to examine, among treatment-seeking individuals with current BN: i) the proportion of those with an AN history (AN+); ii) whether AN+ patients differed from those without an AN history (AN-) in terms of eating disorder (ED) or general psychopathology; and iii) whether AN+ and AN-patients responded differently to CBT-E, in terms of symptoms reduction and attrition rates. Participants were 221 outpatients at the Centre for Clinical Interventions (CCI) Eating Disorders Service, WA. All participants were ≥16 years and met DSM-IV criteria for BN. Results showed that initially, AN+ patients had significantly lower BMI and a longer time since first ED diagnosis. The groups did not differ on levels of general psychopathology at presentation, rates of attrition or in the proportion achieving BN remission. Multilevel linear modelling showed that frequency of binge eating, vomiting and laxative misuse, and scores for EDEQ, perfectionism, depression, anxiety, stress and distress tolerance all improved significantly over treatment, but there was no difference improvement between AN+ and AN-patients. Results suggest that a history of AN does not detrimentally affect individuals' rates of recovery from BN or improvement in mental health factors, and supports a transdiagnostic approach to treatment.

